# Training With Simulated Scotoma Leads to Behavioral Improvements Through at Least Two Distinct Mechanisms

**DOI:** 10.1167/iovs.64.1.14

**Published:** 2023-01-19

**Authors:** Mandy K. Biles, Marcello Maniglia, Ishant S. Yadav, Jason E. Vice, Kristina M. Visscher

**Affiliations:** 1 Department of Psychology, The University of Alabama at Birmingham, Birmingham, Alabama, United States; 2 Department of Psychology, The University of California at Riverside, Riverside, California, United States; 3 Department of Neurobiology, The University of Alabama at Birmingham, Birmingham, Alabama, United States; 4 School of Optometry, The University of Alabama at Birmingham Heersink School of Medicine, Birmingham, Alabama, United States

**Keywords:** peripheral vision training, acuity, crowding, preferred retinal locus (PRL)

## Abstract

**Purpose:**

Individuals with central vision loss due to macular degeneration (MD) often spontaneously develop a preferred retinal locus (PRL) outside the area of retinal damage, which they use instead of the fovea. Those who develop a stable PRL are more successful at coping with their vision loss. However, it is unclear whether improvements in visual performance at the PRL are specific to that retinal location or are also observed in other parts of the retina. Perceptual learning literature suggests that the retinal specificity of these effects provides insight about the mechanisms involved. Better understanding of these mechanisms is necessary for the next generation of interventions and improved patient outcomes.

**Methods:**

To address this, we trained participants with healthy vision to develop a trained retinal locus (TRL), analogous to the PRL in patients. We trained 24 participants on a visual search task using a gaze-contingent display to simulate a central scotoma.

**Results:**

Results showed retinotopically specific improvements in visual crowding only at the TRL; however, visual acuity improved in both the TRL and in an untrained retinal locus.

**Conclusions:**

These results suggest that training with an artificial scotoma involves multiple mechanistic levels, some location-specific and some not, and that simulated scotoma training paradigms likely influence multiple mechanisms simultaneously. Eye movement analysis suggests that the non-retinotopic learning effects may be related to improvements in the capability to maintain a stable gaze during stimulus presentation. This work suggests that effective interventions promoting peripheral viewing may influence multiple mechanisms simultaneously.

Perceptual learning, the improvement in perceptual abilities based on repeated practice, is a widely studied topic in vision research.^1^ Evidence of location-specificity and stimulus feature-specificity of perceptual learning led researchers to believe perceptual learning effects were due to changes in low level perceptual areas.^2^ However, more recent studies showed that elements of the training regime can be manipulated to obtain transfer of learning and generalization,^3^^,^^4^ suggesting that perceptual learning can rely on different regions of the brain, even beyond primary perceptual areas.^5^ Thus, patterns of generalization (or lack thereof) can provide insight about the substrates and mechanisms involved in the observed training effects.^6^ For example, ocular specificity would point toward an early neural locus of learning^2^ most likely in early visual areas that include monocular units,^7^ whereas motion direction specificity would suggest changes in medial-temporal areas.^5^ Thus, knowing what perceptual changes occur can help identify which brain changes are likely to have given rise to the effect.

In more recent years, perceptual learning has benefitted from the potential generalization of learning by successfully treating a number of visual pathologies such as myopia,^8^^,^^9^ presbyopia,^10^ and amblyopia.^11^^,^^12^ Initial attempts have been made to use perceptual learning as a clinical intervention for more severe visual pathologies, such as those resulting in central vision loss like macular degeneration (MD), which currently represents a serious health concern worldwide.^13^^–^^16^ Individuals in the late stages of MD experience complete central vision loss due to progressive degeneration of the most central part of the retina, which possesses the highest level of visual acuity. As the disease progresses, individuals with MD often lose the ability to perform tasks that require a high level of visual acuity such as reading, recognizing faces, or locating objects in a distracting environment. To compensate for the loss of foveal vision, many individuals with MD develop a specific peripheral retinal region outside the damaged fovea, a preferred retinal locus (or PRL), with which they perform demanding visual tasks, such as reading and recognizing faces.^17^^,^^18^

Although perceptual learning results in MD are moderately encouraging,^19^^–^^21^ research in this clinical population presents a number of drawbacks due to recruitment, comorbidity, heterogeneity of disease, and compliance. To overcome this, some laboratories have started to simulate central vision loss in individuals with healthy vision through the use of an eye tracker-guided, gaze-contingent display.^22^^–^^33^ Despite the differences between simulated and pathological central vision loss, such as the visibility of the occluder and the crucial role of its sharp edges in increasing scotoma awareness,^32^ this framework can be used as a model for MD, allowing for a tightly controlled experimental setting.

Kwon and colleagues^34^ showed that using a gaze-contingent display to simulate central vision loss in individuals with healthy vision can induce some of the oculomotor behaviors observed in individuals with MD, such as the development of a PRL, albeit on a much faster time scale (a few hours of training versus a few months of spontaneous viewing^35^). Similarly, Chen and colleagues^36^ showed that simulating central vision loss induces the “shrinking” of the crowding zone, a phenomenon observed in individuals with MD. Crowding, a common feature in peripheral vision, is a perceptual phenomenon in which the ability to discriminate details in stimuli is impaired by the presence of flanking objects located nearby.^37^ Flankers within a crowding zone impair performance above some threshold level. Crowding is characterized by anisotropy between the radial and tangential directions. The crowding zone is elongated along the radial axis connecting the target with the fovea.^38^^–^^42^ Consequently, the crowding zone appears more elliptical the further the configuration is placed away from the fovea. Individuals with MD show a less elongated area of crowding in their PRL with respect to control participants tested at the same eccentricity, suggesting a “fovea-like” reorganization has taken place in the PRL.^39^ Additionally, Liu and Kwon^43^ showed that gaze contingent paradigms can improve static attention, letter recognition, and reading speed. These behavioral improvements were observed in the trained retinal location (“TRL”) but not in a symmetrical, untrained retinal location (“URL”), suggesting location specificity of learning.

Evidence of improved peripheral visual functions after training with simulated central vision loss, in which the central part of the visual field, including the foveola and fovea, is obstructed by an opaque occluder, offers a promise that peripheral vision training can be a model for MD, and that similar training may be helpful for individuals suffering from central vision loss. It is, therefore, important to understand what mechanisms underlie these improvements in behavior. In this study, we trained participants with healthy vision to use a TRL during a visual search task with gaze-contingent, simulated central vision loss. We tested the effects of this training on visual functions, including crowding and acuity. We included a control retinal location (to test for generalization of learning), and a control participant group (to test for practice effects), unlike most previous studies.

Similar to Liu and Kwon^43^ and classic perceptual learning studies,^1^ we found retinotopically specific improvements in crowding at the TRL. However, we also found significant improvements in visual acuity in both the TRL and the URL. Location-specific changes in crowding point towards an early neural locus.^2^ However, transfer of learning of visual acuity to an untrained region hints toward higher-level processing areas, which are not bound to retinotopy,^3^^,^^4^ or to a different non-retinotopic mechanism. The pattern of results we observed suggests that there may be multiple mechanisms underlying training with a simulated scotoma.

Eye movement analysis during the crowding task showed a significant improvement in the ability to maintain a stable gaze toward a target (here termed “fixation stability”) for the trained group but not for the control group, suggesting that increased oculomotor control might have played a role in the learning effects observed. This is consistent with a recent study in which healthy participants trained with artificial scotoma of varying sizes showed consistent orientation of the PRL across scotoma sizes as well as increased fixation stability.^23^ Indeed, fixation stability in individuals with MD has been linked to visual functions, such as visual acuity and reading.^44^^–^^48^ Similarly, Falkenberg, Rubin, and Bex,^49^ testing participants with healthy vision by jittering single or crowded letters or words presented in peripheral vision, found that crowding and visual acuity are related to fixation stability. Taken along with the previous data, our results suggest that the mechanisms responsible for performance improvements following training with simulated scotoma involve not only retinotopically specific sensory improvements, typical of classic perceptual learning, but also generalization of learning to untrained retinal regions, possibly mediated by refinement of oculomotor control.

## Methods

### Participants

Thirty-three participants (6 men and 27 women), average age 24 years (age range = 18–30 years) were recruited from the campus of the University of Alabama at Birmingham and the greater Birmingham metropolitan area. Participants were randomly assigned to either a training group or control group (9 received no training controls, 12 were trained to use a TRL in the right visual field, and 12 were trained to use a TRL in in the left visual field). For all participants, the location of the TRL was randomly assigned (random permutation of the equal number of overall left/right TRLs). Participants had normal or corrected-to-normal vision with no self-reported cognitive or neurological impairments. Their eye dominance was determined by the Porta test,^50^ in which they were told to hold a cylinder at arm's length and look through it at a distant object. Without moving the cylinder, they closed each eye in turn. The dominant eye was recorded as the eye for which, when used alone, the distant object was still visible. Participants received monetary compensation for their participation. The experimental protocols were approved by the Institutional Review Board (IRB) of the University of Alabama at Birmingham and written informed consent was obtained from all participants prior to the experiment.

### Stimuli and Apparatus

The stimuli were generated and controlled using MATLAB (version 8.4) and Psychophysics Toolbox and EyeLink Toolbox extensions^51^^–^^53^ for Windows 8 or 10 (for the training protocol) and for Ubuntu (a Linux operating system interface) for the assessments. All training and assessments were run on a desktop computer (model: ASUS M38 series). The stimuli were displayed on a 32 inch liquid crystal monitor (Cambridge Research Systems Display++; refresh rate: 120 Hz; resolution: 1920 × 1080) located 57 cm away from a participant's eyes while their head was stabilized in a chin rest. Participants’ eye movements were monitored (monocular tracking with their dominant eye, as determined by the Porta test^50^) using an infrared video-based eye-tracker sampled at 500 Hz (EyeLink 1000 Plus/Desktop Mount, SR Research Ltd., Ontario, Canada). A nine-point calibration/validation sequence was performed at the beginning of every experimental assessment and training block that relied on the eye-tracker. Calibration and/or validation were repeated until the validation error was smaller than 1 degree on average. The gaze position error (i.e. the difference between the target position and the computed gaze position) was estimated during the nine-point validation process.

Outcome measures visual acuity and critical space are reported in degrees of visual angle (dva), which were calculated based on the size of the screen, the number of pixels in the image of the Landolt “C,” and the distance between the eye and the screen (57 cm). A gaze-contingent visual display obstructing central vision was used throughout the training.^32^^,^^43^^,^^54^ A circular gray patch (luminance, 18 cd/m^2^) with a radius of 6 degrees and centered on the tracked location of the fovea, was the “simulated scotoma” used to obstruct central vision. Throughout the training, the real-time gaze position was sent to the display computer through a high-speed ethernet link. During the assessments, participants’ performance was measured at a fixed (gaze-contingent) distance from the fovea. Given that a blink or a squint could transiently cause the reported eye position and location of the simulated scotoma to be different than the actual gaze, our protocol minimized this mismatch by turning the entire display screen blank (gray) as soon as a blink was detected or the pupil size was decreased to a threshold value.^55^ To verify the system latency, the average delay between eye movement and screen update was measured using methods from Saunders and Woods.^56^ Specifically, an LED light was aimed to shine both on the eye tracker camera and was turned off and on during presentation of a slightly altered version of the exogenous attention task while an experienced volunteer performed the task. The LED light disrupted the signal to the eye tracker, causing the display to turn gray. A 240 Hz video recording was made which captured both the screen and the LED light. Measuring the time between LED light and screen disruption using the video showed a screen update of about 25 ms (median value of 50 measurements). Although this measured update time is not as fast as technically possible given our setup, it was fast enough to result in comfortable task performance.

We used different codes to collect data for crowding and visual acuity; the code we developed for crowding was specifically designed for examining eye movements, whereas the code for visual acuity assessment was not. We used the data from the crowding task to evaluate the ability to maintain a stable gaze toward a target. This is measured as the logarithm of the bivariate contour ellipse area (log[BCEA]) of the fixation locations during target presentation, and for brevity it is termed here “fixation stability” because it represents stability of fixation locations. The algorithm for calculating fixation stability is described in more detail in our previous work.^28^ Note that this measure of fixation stability is calculated and averaged across trials, and is therefore distinct from other measures of the stability of fixation, which measure stability over many seconds of constant fixation.

### Training

**Figure 1.**
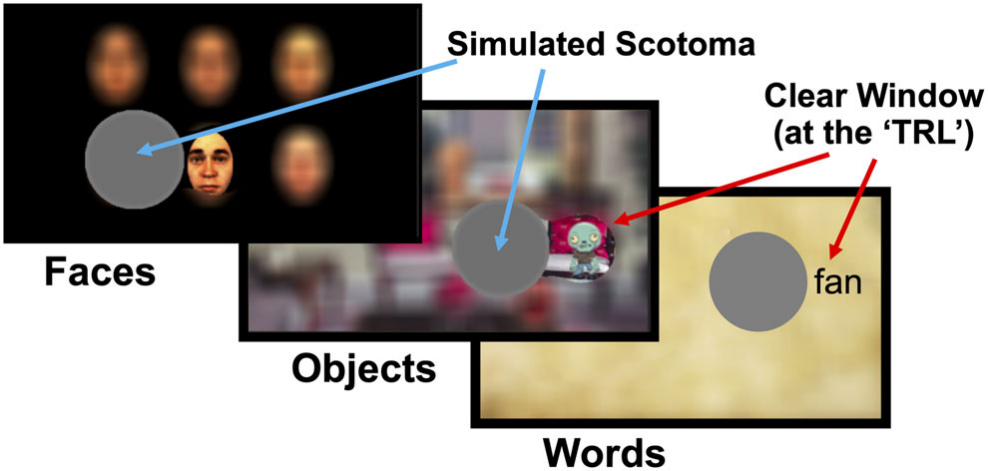
Simulated scotoma paradigm. Example frames are shown for images of faces, objects, and words. The *gray circle* is the simulated scotoma, presented at the center of the participants' gaze (6 degrees radius). To the right of this scotoma, a clear window showed unblurred images (2.5 degrees radius, and centered 8.5 degrees from center of the scotoma) for participants trained to develop their TRL on the right.

The training task used a simulated scotoma protocol described in detail by Liu and Kwon.^43^ The background on the screen was blurred except for the training location, a circular region of clear view centered 8.5 degrees to the right or the left of the center of the simulated scotoma (Fig. 1). Participants were trained in three blocks: face recognition, object recognition, and word recognition. Each time participants came to the laboratory, they were trained on all three blocks. Training blocks consisted of 30 trials each.

**Figure 2.**
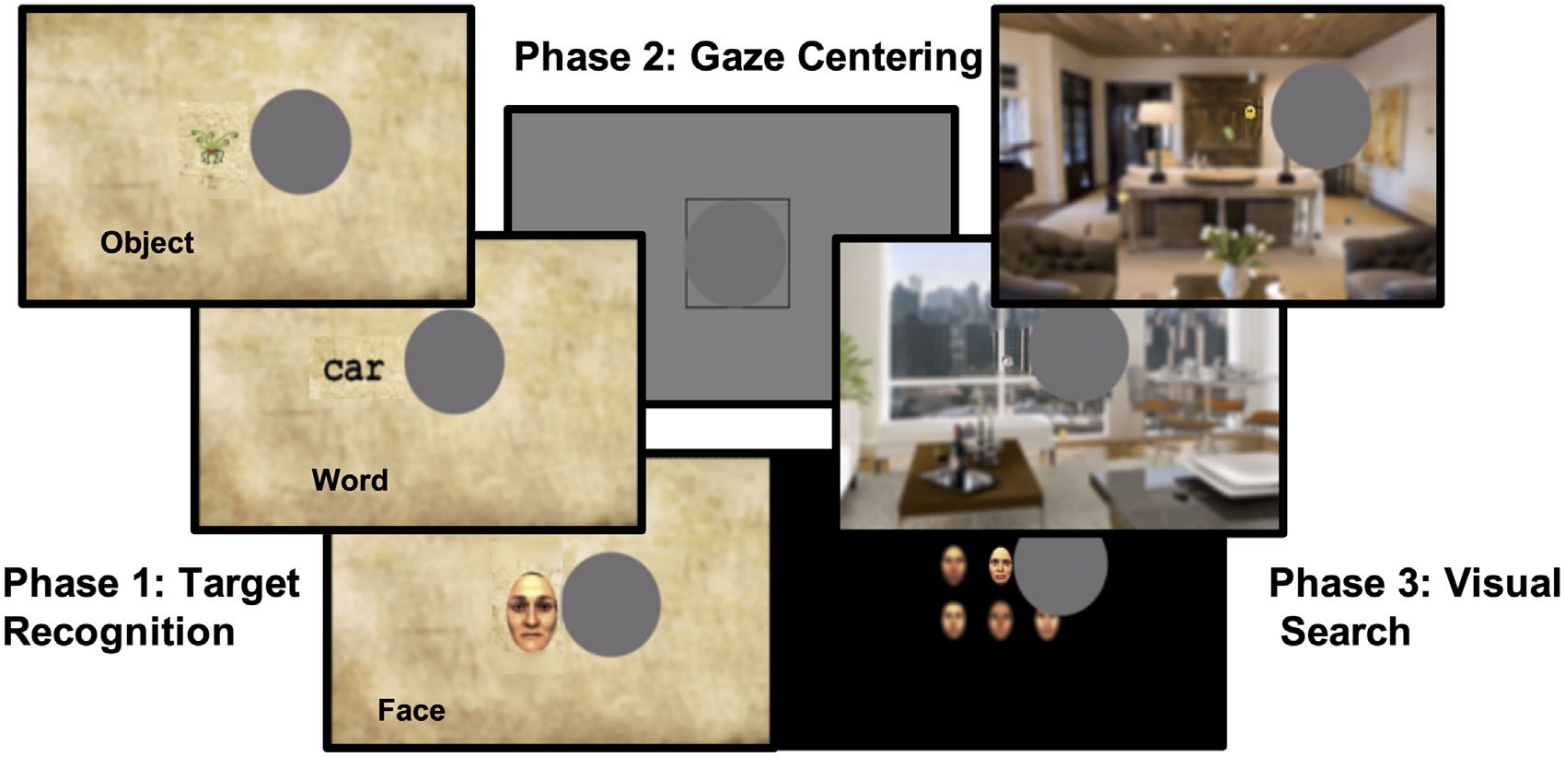
The three phases within the training protocol. Phase 1 was the target recognition phase in which the participant performed a visual search for targets appearing in a parchment background. Stimuli are shown larger for clarity. Phase 2 was the gaze centering phase in which the participant centered their gaze within the *darker gray box*. Phase 3 was the visual search phase in which the participant performed a search of the blurred scene or array of faces to determine if the last image presented during the target recognition phase was present within the scene or array of faces. A gaze contingent display with a clear window (centered at 8.5 degrees with a diameter of 5 degrees) was used to train participants to use a specific trained retinal location (TRL) to perform the tasks. Information from central vision was covered by a simulated scotoma, whereas the rest of the screen, except the clear window, was blurred. Tasks involved searching for and identifying faces, words, or objects.

Each trial had three phases: target recognition, gaze centering, and visual search (Fig. 2). During the target recognition phase, targets (face, word, or object) were presented on a blurred parchment background and participants were asked to position the clear view window or TRL over the target and then identify each target as either a male or female face in the face recognition block, an object or a cartoon monster in the object recognition block, or a word or random letter string in the word recognition block. Within the target recognition phase, there were five trials followed by a sixth trial in which the stimulus presented was the target stimulus for the visual search phase. Participants were then asked to center their artificial scotoma in a black box in the center of the screen during the gaze centering phase in order to minimize any positional bias during the visual search phase. Finally, participants were instructed to report whether the target stimulus from the final trial in the target recognition phase was present or absent amidst either a solid black background (face search task) or a blurred scene of a room, such as a living room or kitchen, with an array of non-target distracters (word search task or object search task) during the visual search phase.

Trained participants were exposed to all three training blocks at each training session. Training sessions each took approximately 45 minutes to 1 hour to complete. Over the course of 4 to 6 weeks, participants completed a total of 12 sessions of training. The order in which participants completed the training blocks at each session was randomly assigned before the first training session and the block order was kept the same for all training sessions for each participant. Participants were randomly assigned to a TRL or to the control group before the baseline assessment session. The participants in the control group were only required to attend baseline and post assessment sessions. During the 4 to 6 weeks between baseline and post assessment sessions, while the trained participants came to the laboratory for the 12 training sessions, the participants in the control group received no training.

### Visual Acuity

**Figure 3.**
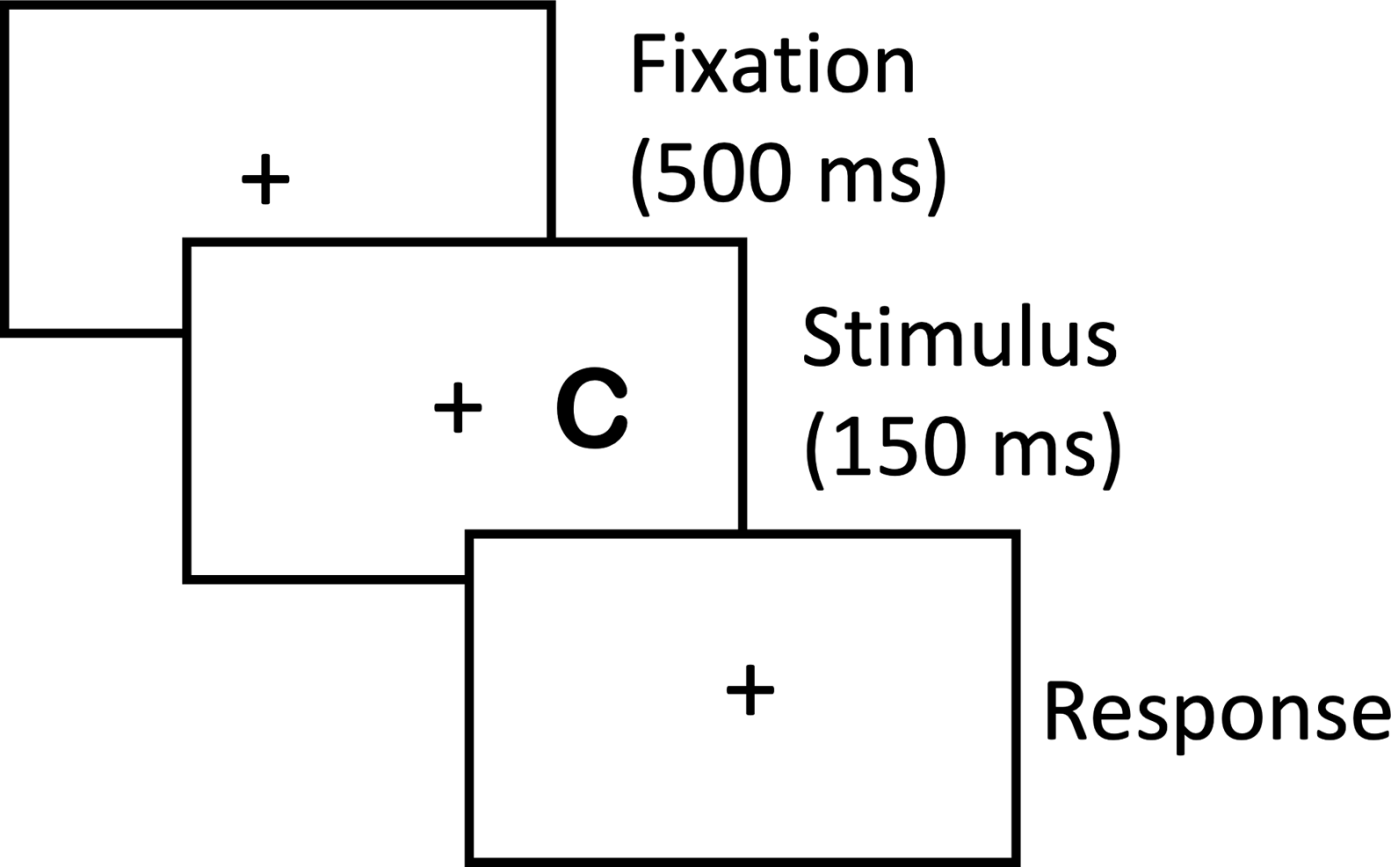
Procedures for measuring visual acuity. The participants' task was to report the orientation of the gap in the Landolt “C” by pressing one of four arrow keys on a standard keyboard.

Visual acuity was measured before the first training session and after the last training session using a gaze-contingent display and using the method of constant stimuli with Landolt “C”s (Fig. 3). Each trial started with a 500 ms fixation display, followed by a Landolt “C” presented for 150 ms at 8.5 degrees eccentricity either to the right or left (TRL and URL for individuals trained on the right) of the fixation cross and rotated 0 degrees, 90 degrees, 180 degrees, or 270 degrees (randomized for each trial). The stimulus letter was black against a uniform white background. The participant was asked to report the orientation of the opening in the Landolt “C” by pressing one of the four arrow keys on a keyboard. Participants were tested on 5 stimulus letter sizes, spanning a range of 0.9 log units. Trials of each stimulus size were interleaved randomly; each stimulus size was presented 20 times for a total of 100 trials divided in 2 blocks per side (TRL and URL) for a total of 4 runs of 50 trials each per side. Threshold, defined as 80% correct recognition was calculated for each block. Blocks of trials were sequenced in order to counterbalance the order of blocks. Visual acuity at the TRL at baseline is reported based on the average threshold for each of the two blocks performed at the TRL. Visual acuity at the URL, and at post-test are reported analogously.

### Visual Crowding

**Figure 4.**
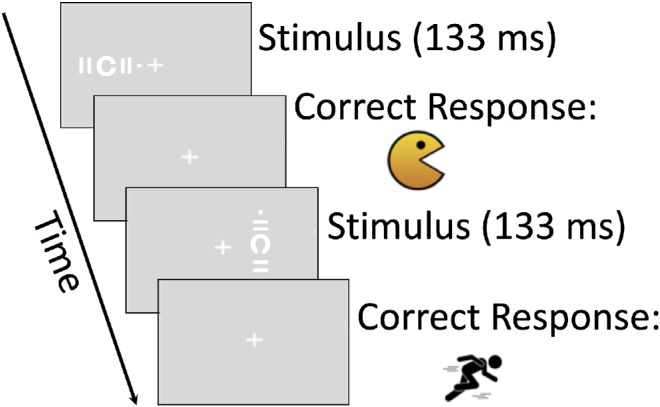
Procedure for the crowding task. The stimulus presentations for the crowding task consisted of a Landolt “C” surrounded by flanking bars on each side of the letter in either the radial or tangential orientation with respect to the central fixation cross. A dot was situated on the right side of the flanking bars when the stimulus was presented horizontally and on the top when the stimulus was presented vertically. After stimulus presentation, the participant responded by pressing a button corresponding to “eating” the dot when the opening of the “C” was oriented in the direction of the dot or “running away” when the opening of the “C” was oriented away from the location of the dot.

Crowding was measured before the initial training session and after the last training session. The stimuli consisted of a Landolt “C” surrounded by two lines (flanking bars) on each side of the letter (Fig. 4). A dot was situated on the right side of the flanking bars when the stimulus was presented horizontally and situated on the top when the stimulus was presented vertically. Participants were asked to maintain fixation on a centrally located fixation cross and were then presented with the stimulus at 8.5 degrees eccentricity either to the left or to the right of the fixation cross (TRL and URL). After each presentation, participants were asked to determine the orientation of the opening of the Landolt “C” by reporting whether the “C” was “eating” the dot or “running away” from the dot by pressing the button on the button box with images that corresponded with “eating” (chomping monster) when the opening of the “C” was oriented in the direction of the dot or “running away” (running man) when the opening of the “C” was oriented away from the location of the dot. Participants were given auditory feedback on their performance after each response (tone indicating correct or incorrect). The critical space of crowding (the edge-to-edge distance between the “C” and the bars) varied according to a 3:1 staircase, for which an incorrect response leads to an increase and 3 consecutive correct responses lead to a decrease of critical space leading to a 79% accuracy. To minimize known issues with this procedure, in particular the accuracy in reaching the targeted performance level, we used adaptive step sizes decreasing every reversal and a relatively high number of trials (100) or reversals (16) to compute the threshold. The initial distance between the Landolt “C” and the flanking bars was set at 2.3 degrees visual angle, and the smallest step size was 0.01 log units. The stimuli were presented for 133 ms. As in the case of the visual acuity task, stimulus location was randomized between TRL and URL on a trial basis. The critical space of crowding was estimated by averaging the thresholds of the last eight reversals. Two separate staircases were interleaved to estimate crowding along the radial and tangential directions. We then calculated the anisotropy between radial and tangential directions,^38^^–^^42^^,^^57^ as the ratio (radial threshold/ tangential threshold).

## Results

To examine the effect of peripheral vision training on low-level visual functions, we measured visual acuity and visual crowding at the TRL and at a symmetrical, URL before and after training and tested for location-specific effects at the TRL and URL. Performance improvements were quantified as the change in the post-training threshold with respect to the baseline threshold for each location independently. A value of zero would indicate no change between baseline and post-training, whereas a number greater than zero would indicate improvement after training. Paired *t*-tests were used to assess training effects.

### Visual Acuity

One trained participant was excluded from this analysis based upon a post training assessment score that was over three standard deviations from the mean (3.8 SD) and one control participant did not have data for this task due to a technical error. Numbers of participants included in this analysis were therefore: control = 8 and trained = 23. ^1^

**Table:** 

Acuity Analysis	df	F-Stat	*P* Value	Eta
**Session**	1,29	6.76	0.015	0.189
**Group**	1,29	0.163	0.69	0.006
**Location**	1,29	0.092	0.76	0.003
**Session X group**	1,29	6.33	0.018	0.179
**Session X group X location**	1,29	0.02	0.889	0.001
**Group X location**	1,29	2.07	0.16	0.067

**Figure 5.**
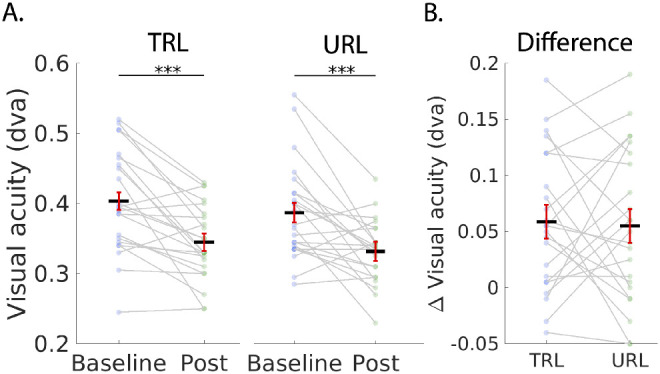
Visual acuity threshold results for the TRL and the URL in the trained group. (**A**) Baseline and post training comparisons for the TRL and the URL in terms of degrees visual angle (dva). (**B**) Performance improvements for the TRL and the URL showing that the significant training gains for both the TRL and the URL were not significantly different from each other. *Dark lines* represent means across subjects. Data points from individuals are connected with gray lines. Error bars are 95% confidence intervals.^60^ Stars indicate ***P < 0.001.

We found no evidence for a baseline difference in visual acuity between locations for either the trained or the control groups. For the trained group the baseline TRL versus URL analysis (2-tailed), t(22) = 0.93, *P* < 0.36, Bayes factor = 0.32, which is interpreted as “substantial” evidence for the null hypothesis.^58^ For the control group t(7) = −0.76, *P* < 0.47, Bayes factor = 0.43, “marginal” evidence for the null hypothesis.^58^ Similarly, no left versus right baseline difference was observed: (left versus right TRL: t(29) = 0.11, *P* = 0.913; left versus right URL: t(29) = 1.68, *P* = 0.103). In our location specific analysis, paired *t*-tests showed a significant reduction in visual acuity thresholds for the trained group both in the TRL t(22) = 4.60, *P* = 7.00 × 10^−5^, Bayes factor = 403.2 and in the URL t(22) = 3.88, *P* = 8.16 × 10^−4^, Bayes factor = 84.61. However, the difference between the TRL and URL for the trained group was not significant t(22) = −0.24, *P* = 0.59, Bayes factor = 0.18, “substantial” evidence for the null hypothesis.^58^ These results suggest that the training effect was not retinotopically specific to the TRL (Fig. 5^6^). Additionally, we compared training gain left versus right, which showed no lateralization effects (trained group, left versus right TRL gain: [2-tailed], t(22) = 0.202, *P* = 0.841).

**Figure 6.**
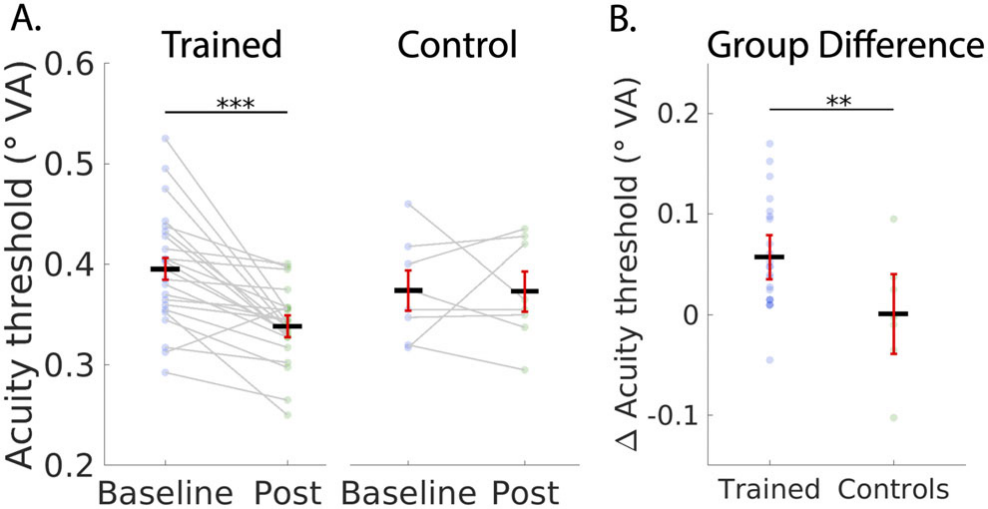
Visual acuity threshold results for the trained and control groups. (**A**) Baseline and post training comparisons for the trained and control groups. *Dark lines* represent means across subjects and data points from individuals are connected with *gray lines*. Error bars are 95% confidence intervals.^60^ (**B**) Performance improvements for the trained and control groups showing that the trained group had significant training gains compared to controls. Error bars are 95% confidence intervals for the group difference. Stars indicate ***P < 0.001 and **P < 0.01.

To test for location nonspecific effects of training, we followed up with a test directly comparing the trained group and control group (Fig. 6). In these analyses, because there was no significant difference between the TRL and the URL, we averaged the scores at the two locations and compared the performance gain between groups. In our group level analysis, paired *t*-tests showed a significant improvement in visual acuity after training for the trained group t(22) = 5.13, *P* = 1.93 × 10^−5^, Bayes factor = 1284 but not for the control group t(7) = 0.046, *P* = 0.48, Bayes factor = 0.35. The between groups difference of the baseline – post-training thresholds was also significant t(29) = 2.52, *P* = 0.0088, Bayes factor = 6.59.

Of note, these visual acuity thresholds are not dissimilar from those measured in a previous study^28^ that used a similar paradigm (Pre = 0.648 logMAR [SEM = 0.043], Post = 0.523 logMAR [SEM: 0.021] versus Pre = 0.62 logMAR [SEM = 0.034], Post = 0.582 [SEM = 0.031] here).^2^

### Crowding

#### Crowding Radial/Tangential Ratio

Concerning crowding, our hypothesis was that the trained participants would have a reduced crowding ratio (crowding extent measured radially and tangentially with respect to the fovea) after training, and that this effect would be specific to the TRL. We therefore performed mixed model ANOVA with between factor group (trained versus control) and within factors location (TRL versus URL) comparing the (log) crowding ratio at the TRL and URL at baseline versus after training. The critical test of the hypothesis was a *t*-test comparing the effects of training at the TRL versus the URL. The results of the mixed model ANOVA are shown in the table, indicating a significant interaction of session X group X location.

**Table:** 

Crowding Ratio Analysis	Df	F-Stat	*P* Value	Eta
**Session**	1,31	0.878	0.356	0.028
**Group**	1,31	0.161	0.691	0.005
**Location**	1.31	0.012	0.912	0.001
**Session X group X location**	1,31	4.263	0.047	0.121

**Figure 7.**
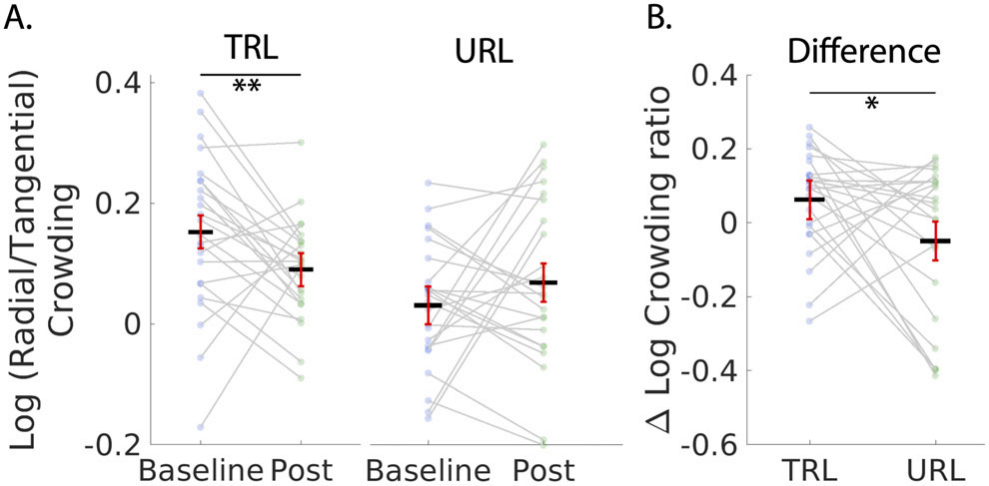
Logarithm of the radial/tangential crowding ratios for the TRL and the URL in the trained group. (**A**) Baseline and post training comparisons for the TRL and the URL. *Dark lines* represent means across subjects. Data points from individuals are connected with gray lines. (**B**) Performance improvements (measured as baseline - post) for the TRL and the URL showing significant training gains for the TRL compared to the URL. Error bars are 95% confidence intervals.^60^ Stars indicate a significant result (*P < 0.05 and **P < 0.01).

The critical test of our hypothesis that the training effect would be specific to the TRL compared the difference between TRL and URL in the trained group. In order to perform statistics on these ratio data, the data were transformed by a log_10_ of the ratio. Paired *t*-tests of data in the trained group showed a significant reduction in the radial/tangential ratio in the TRL t(23) = 2.59, *P* = 0.0081, Bayes factor = 6.41. The crowding ratio was not significantly reduced in the URL t(23) = −1.46, *P* = 0.16, Bayes factor = 0.086 (Fig. 7A). Critically, the difference between the TRL and URL was significant t(23) = 2.46, *P* = 0.011, Bayes factor = 5.05 indicating substantial evidence^58^ that the effect of training on the radial/tangential ratio is retinotopically specific (Fig. 7B).

### Fixation Stability and Oculomotor Metrics Analyses

Results from our low-level assessments indicate that the training reduced visual crowding at the TRL. Additionally, we saw improvements in visual acuity, but these improvements were not location specific – they were observed for the URL as well. Although it is common in perceptual learning to observe transfer of learning to more complex tasks when participants are trained on low level components,^1^^,^^10^ it is less common to find a transfer from “high-level” tasks, such as object and face recognition to low level visual abilities, such as visual acuity, especially in an untrained retinal location as is the case here. This led to the hypothesis that visual acuity was improving due to a change in visual input.

To test this hypothesis, we examined fixation stability. Poor stability leads to non-stationary visual inputs, which are likely to be less well processed. We measured fixation stability during the crowding task, during which we had collected the appropriate data for this analysis. In particular, because our hypothesis was that a steadier fixation might have better stabilized the image of the target on the retina, we looked at the time interval corresponding to target presentation (150 ms). Following previous studies,^45^ we expressed fixation stability the square root of the bivariate contour ellipse area (log BCEA degree^2^)^59^ encompassing 68% of the overall eye position in that time window. In this framework, a small BCEA indicates more stable fixation. The BCEA scores had a non-normal distribution, with a long tail. In order to decrease the effect of this long tail on analyses, we performed all analyses on the log of the BCEA, rather than the raw value. Units are degrees; the log of the BCEA is proportional to the effective radius of the eye position distribution. A mixed model ANOVA with time (Pre versus Post) as a within-factor and group (trained versus control) as a between factor showed no significant main effects or interactions (time: F(1,31)= 2.783, *P* = 0.105, eta = 0.082, interaction: F(1,31) = 2.035, *P* = 0.164, eta = 0.062, group: F(1,31) = 0.0635, *P* = 0.800, eta = 0.002). With outlier removal: (time: F(1,29)= 3.319, *P* = 0.84, eta = 0.099, interaction: F(1,29) = 1.64, *P* = 0.210, eta = 0.054, group: F(1,29) = 0.128, *P* = 0.723, eta = 0.004).

However, paired *t*-tests conducted separately for each group showed a significant improvement in fixation stability for the trained group t(23) = 2.615, *P* = 0.015, Bayes factor = 9.43 but not the control group t(8) = 0.33, *P* = 0.75, Bayes factor = 0.55.

**Figure 8.**
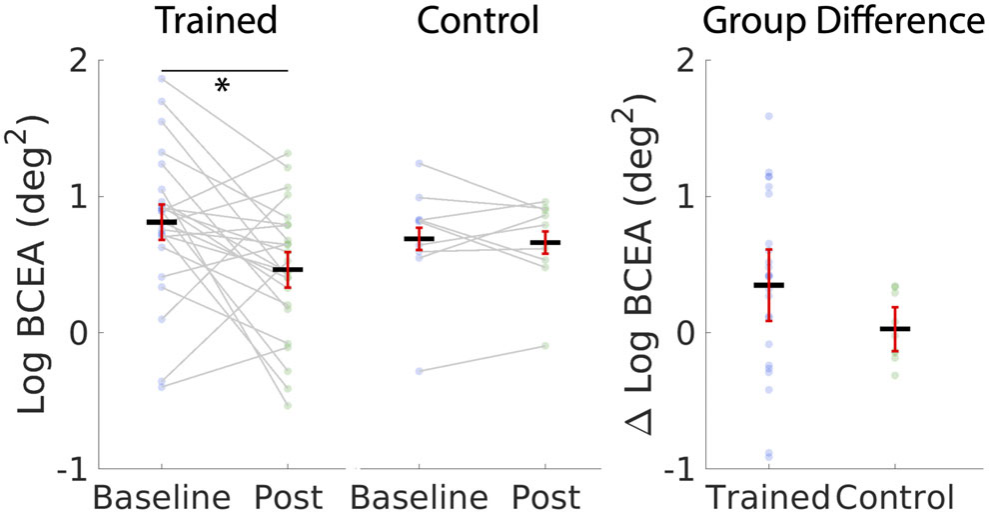
(**A**) Fixation stability results. Fixation stability (expressed as the square root of the bivariate contour ellipse area corresponding to 68% of the overall eye positions during target presentation in the crowding task)^59^ before and after training for the trained and the control groups. Fixation stability significantly improved in the trained group, but not in the control group. *Dark lines* represent means across subjects. Data points from individuals are connected with *gray lines*. (**B**) Difference in fixation stability after training for the trained versus the control group. Note that the effect of training was trending in the hypothesized direction (*P* = 0.082), but was not significant. Error bars are 95% confidence intervals.^60^ *Stars* indicate a significant result (**P < 0.01).

The mean training gain was numerically larger for the trained group than the control group, as would be expected (Fig. 8), but this difference was not statistically significant t(31) = 1.43, *P* = 0.082, Bayes factor = 1.39.

**Figure 9.**
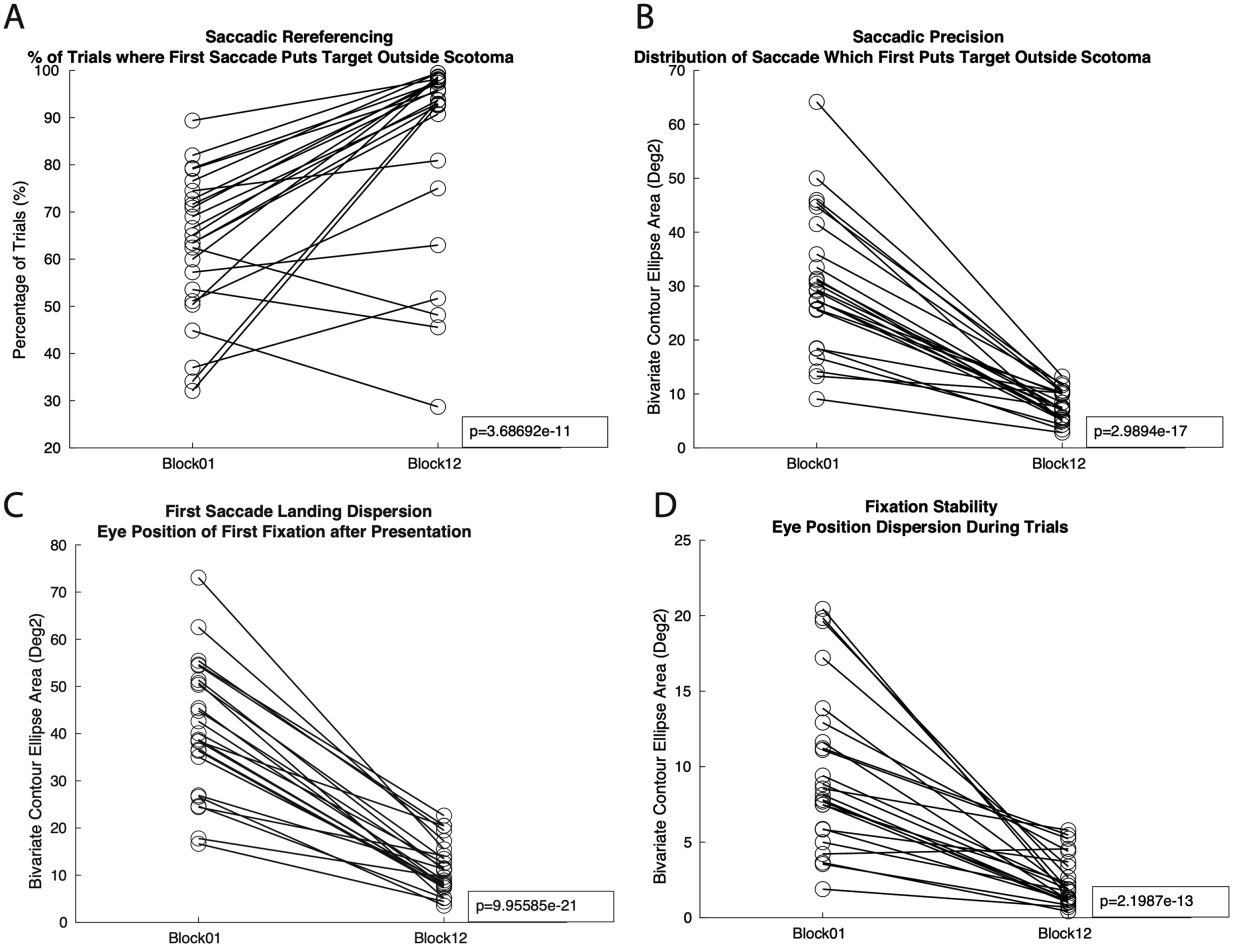
(**A**) Evidence that peripheral vision training did improve participants’ gaze toward peripheral targets. Each linked pair of points represents a participant's performance at the beginning of training (block 01), to the end of training (block 12) for each of the four oculomotor metrics: (**A**) saccadic re-referencing, (**B**) saccadic precision, (**C**) first saccade landing dispersion, and (**D**) fixation stability.

Further eye movement analyses were conducted adopting some of the oculomotor metrics described in Maniglia, Visscher, and Seitz (2020) and reported in Figure 9. These metrics are intended to characterize compensatory oculomotor behavior following (simulated) central vision loss along several metrics, beyond the classic fixation analysis (e.g. Crossland et al., 2004). that are relevant in the context of eye movement. In particular, we focused on four of those metrics, namely saccadic re-referencing (Fig. 9A), which looks at (1) the percentage the time that the first saccade after target appearance places the target outside the scotoma (a higher percentage of first saccades not covering the scotoma would suggest a higher degree of oculomotor re-referencing away from the fovea); (2) saccadic precision, which quantifies the dispersion (in degrees^2^) of the landing location of the first saccade that places the target in a visible location outside the scotoma (Fig. 9B); (3) first saccade landing dispersion (Fig. 9C), which measures the landing area of the first saccade of the trial; and (4) fixation stability (Fig. 9D). Results showed that, when comparing the first with the last day of training, participants in the trained group significantly improved in all the oculomotor metrics described above (all *P* < 0.001). Taken together, results from these eye movement analyses suggest that, alongside post-training improvements in fixation stability observed during the crowding task, participants increased their control of peripheral vision after training.

## Discussion

In the present study, young participants with healthy vision were trained to use a specific region in the peripheral visual field (TRL) to perform visual tasks during gaze-contingent, simulated central vision loss. Consistent with the literature on perceptual learning (see Sagi and colleagues^1^), we did find a retinotopically specific reduction in crowding at the TRL. However, we also observed improvements in visual acuity in both the TRL and in a symmetrical, untrained region (URL), and there is substantial evidence for the null hypothesis that the two are not different. Given that perceptual learning studies suggest that the amount of transfer (improvements observed in untrained portions of the visual field) is indicative of the neural level at which training-induced learning has taken place,^6^ this difference in specificity suggests that at least two mechanisms underlie the effects observed here. Analysis of eye tracking data does not contradict the idea that acuity improvements may result from a mechanism involving improvements in fixation stability. Indeed, fixation stability seems to be related to peripheral visual acuity and reading, both in participants trained with simulated scotoma^36^ and individuals with MD.^44^^,^^46^^–^^48^^,^^61^ Moreover, it has been proposed that reading difficulties may depend on unstable fixation,^49^ which may benefit of rehabilitative interventions based on improving fixation stability.^49^

Taken as a whole, our results suggest that in training involving the use of simulated scotoma,^22^^,^^26^^–^^28^^,^^31^^,^^32^^,^^34^ there are likely at least two mechanisms underlying improvements in visual behavior. These multiple mechanisms of improvement may carry over to individuals with MD, who are likely to benefit from training approaches which address multiple domains.

Specifically, we observed retinotopically specific improvements in visual crowding, consistent with classic perceptual learning studies where training a single retinal location results in location-specific improvement.^2^ Additionally, our pattern of results, in which the shape of the crowding zone in the TRL changed, is consistent with results from other studies that used training with simulated scotoma.^36^ This is also in line with what is observed in individuals with MD, where those with a developed PRL show a more “symmetrical” shape of the crowding zone (more reduced along the radial axis) than controls with healthy vision tested at the same eccentricity.^39^ Because crowding areas measured at or near the fovea show a less elliptical shape, the interpretation of this evidence in individuals with MD is that the PRL becomes more “fovea-like” due to plasticity.^39^^,^^62^

The neural mechanisms underlying crowding are still debated, with some authors proposing that crowding occurs when elements are grouped into wholes, a process reflected in electroencephalogram (EEG) by the N1 component,^63^^–^^65^ whereas others suggesting earlier neural loci, such as V1 or V2.^66^^–^^68^ Levi^37^ proposed a multistage model of crowding that includes both the detection of simple features (early lateral interactions) and the integration of features downstream from V1. Recent work confirms that changes in crowding might rely on neural changes within V1, where patterns of neural responses indicate less suppression of flanking stimuli following training.^36^ The idea that changes in crowding rely on V1 modifications is also consistent with some behavioral evidence, such as the observed improvement in crowding following lateral masking training,^68^ whose anatomic substrate is thought to be long-range connections found in primary visual cortex.^69^^–^^71^ Contemori and colleagues showed that occipital transcranial random noise stimulation (tRNS) led to a significant boost in crowding reduction training,^72^ suggesting that early visual areas are involved in crowding reduction.^21^ These data from previous studies, along with the finding that the observed changes in crowding were specific for the trained retinal region, suggest that the neural mechanisms supporting the changes in crowding observed here likely result from changes in early visual areas.

In contrast to many perceptual learning studies, we included a no-training control group, allowing us to observe some training-related improvements that were not specific to the trained retinal region. Specifically, we saw improvements in acuity in both the TRL and the URL, which were significantly greater in the trained group than the control group. This pattern of results suggests the involvement of mechanisms located beyond the retinotopically mapped visual areas. It is worth noting that a previous paper using a similar training protocol^43^ did not report an effect on visual acuity as they did not include a no-training control group in their experimental design and only compared the TRL to the URL.

There are a number of possible reasons to account for this generalization of learning. One possibility is that training effects ‘transfer’ to another retinal area because the training relies on higher order brain circuits.^3^^,^^73^^–^^75^ It has been suggested that training and stimulus features are primary predictors of transfer of learning.^6^ For example, repeated training at threshold shows retinotopic specificity, whereas training with alternating “easy” and “difficult” trials generalizes to other locations,^76^ or training participants on one task in one retinal location and subsequently training them on a different task in a different retinal location results in transfer of learning of the first task to the second location.^3^^,^^4^ Although the simulated scotoma training performed here was quite complex and involved higher order stimuli like faces and words, acuity is a very low-level feature. It would be unlikely that acuity would improve due to changes in higher-order cortical regions. Thus, it seems unlikely that our results stem from transfer of learning. An alternative reason that training could be nonspecific is through changes in stimulus input following training. One way to change stimulus input is through changes in eye movements.

A number of studies show evidence relating fixation stability and peripheral visual acuity and reading, both in individuals with MD^45^^–^^48^^,^^77^ and participants trained with a simulated scotoma.^36^ For example, Crossland and colleagues found that in individuals with newly developed MD, small changes in fixation stability over the first 12 months of disease progression are related to changes in reading speed.^45^ Tarita-Nistor and colleagues showed that visual acuity is positively correlated with fixation stability.^47^ Additionally, learning studies suggest that better control over fixation is linked to improved processing of briefly presented visual stimuli,^78^^,^^79^ potentially leading to greater learning and transfer. Indeed, in optometry practice, exercises of guided eye movements improve fixation stability. Morales et al. found improvements in eccentric fixation stability using biofeedback fixation training in microperimetry, when the fixation training locus is individualized as the retinal area with best functional characteristics,^80^ whereas Verdina and colleagues found that biofeedback rehabilitation seems to improve visual acuity, reading performances, contrast sensitivity, retinal fixation and sensitivity, and quality of life in individuals with MD.^81^

To address the possibility that the improvements in acuity in our participants could result from improvements in eye movements, we examined participants’ ability to maintain steady fixation in one of the assessment tasks before and after training. We found that trained participants significantly improved fixation stability after training, and increased their efficiency to use peripheral vision, as assessed by a series of oculomotor metrics developed by Maniglia, Visscher, and Seitz (2020) to characterize use of peripheral vision in conditions of central vision loss. This suggests that the improvements in visual acuity we observed might be related to increased fixation stability and, in general, better use of peripheral vision, following training. However, this interpretation is tempered by the fact that although trained participants showed a statistically significant improvement whereas control participants did not, the difference between groups was not statistically significant. This pattern of results is also consistent with the hypothesis that reading difficulties in peripheral vision may result from unstable peripheral fixation.^49^ Further work concentrating on training-related changes in fixation stability is needed to fully address its relationship to improvements in peripheral acuity.

There are limitations to keep in mind when interpreting these data. Our experiment focused on testing acuity and crowding after training, finding improvements in both, but only crowding was retinotopically specific. In an effort to understand what mechanisms might be different between the two cases, based on literature suggesting crowding was involved, we then examined fixation stability. Our data are consistent with the idea that changes in fixation could drive the observed changes in acuity, but further work is needed to directly test that point. Our untrained control group was relatively small, limiting our power to compare the two trained groups to the untrained group. Further, our data were collected in young adults, whereas the majority of individuals who have macular degeneration are older; characteristics of learning may be different in the two populations, so transfer of this work to relevant therapies should involve tests in older adults.

These data show that training to use peripheral vision likely targets multiple mechanisms of plasticity. Future work developing training protocols for individuals with MD should keep this in mind; the current work suggests that training that incorporates both oculomotor improvements and perceptual learning may be beneficial. In summary, a visual search and object recognition training with a gaze-contingent display simulating central vision loss improves crowding in a location-specific way, and visual acuity in a location nonspecific way. Because there are both location-specific and location nonspecific effects, we conclude that simulated scotoma training involves at least two distinct neural mechanisms. The hypothesis that steadier fixation led to the observed improvements is consistent with both clinical and simulated central vision loss literature and provides insights into the mechanisms of learning in studies adopting simulated central vision loss. These results may be helpful in developing visual training for individuals with MD in which both sensory and oculomotor components are engaged to maximize learning effects.
